# Editorial: Radiofrequency Ablation as an Alternative to Conventional Treatment

**DOI:** 10.3389/fendo.2022.883809

**Published:** 2022-04-27

**Authors:** Loredana Pagano, Cosimo Durante, Ralph Patrick Tufano

**Affiliations:** ^1^ Division of Endocrinology, Diabetology and Metabolism, Department of Medical Sciences, University of Turin, Turin, Italy; ^2^ Department of Translational and Precision Medicine, Sapienza University of Rome, Rome, Italy; ^3^ Department Head and Neck Endocrine Surgery, Sarasota Memorial Health Care System, Sarasota, FL, United States

**Keywords:** radiofrequency ablation (RFA), thyroid nodule, papillary microcarcinomas (PMC), thyroid volume, minimally invasive technique

Ultrasound (US)-guided radiofrequency ablation (RFA) is a minimally invasive treatment modality that may be an alternative to surgery in patients with benign thyroid nodules. In addition, it may serve as an alternative treatment for carefully selected papillary microcarcinomas (PMC) and recurrent thyroid cancers.

In a systematic review on 17 retrospective studies, Monpeyssen et al. provided evidence for the effectiveness of RFA in reducing nodular volume and compressive and cosmetic symptoms in benign thyroid nodules, without causing thyroid dysfunction or life-threatening complications. Indeed, RFA is a percutaneous treatment that results in thermal tissue necrosis and ultimately fibrosis within the target nodule. As a result of this process, the nodules shrink with a 12-month volume reduction rate ranging from 67 to 75% for those lesions undergoing a single procedure. Thermal ablation, however, is an operator-dependent technique and should be performed in centers with RFA specific expertise. Two single center, retrospective studies (Bernardi et al., Bisceglia et al.) found that efficacy should always be evaluated at specific time points (1, 3, 6 and 12 months), with the one-year follow-up visit being generally considered optimal for assessing final outcome. In these cohorts of patients, there was a good association between initial ablation ratio and volume reduction ratio (VRR) 1 and 5 years after the procedure. Proper selection of the patients appears to be a key step with the nodule size (<25ml) and the echotexture (i.e., macrocystic pattern) being the parameters that positively predict the outcome of the treatment.

Moreover, two reviews (Cesareo et al., Pace-Asciak et al.) demonstrated that RFA can be successfully used to treat autonomously functioning thyroid nodules displaying signs or symptoms of compression to adjacent structures, hyperthyroidism, and in pretoxic nodules. Such an approach represents a valuable alternative to therapies that may complicate pre-existing chronic disorders in elderly patients, or that are controversial in young women, like radioactive iodine therapy. Even in these cases, patient selection is essential to optimize treatment efficacy in terms of volume reduction and thyroid function normalization.

Indications for RFA in treating small primary differentiated thyroid cancers is a hot topic now. Benefits on health-related quality of life (HRQL) have been reported in a large cohort of patients with nonaggressive thyroid malignancies not amenable to surgery due to the presence of co-morbidities (Lan et al.) Mauri et al. reported promising data in properly selected, very low risk thyroid cancer patients, especially in PMC, T1 N0, in whom RFA turned out to be a safe alternative to either surgery or active surveillance. RFA could contribute to reduce the treatment burden of surgery and/or the psychological burden of active surveillance for these patients.

The purpose of discussing these issues in the Research Topic of Frontiers in Endocrinology session is to highlight the current and possible future utility of RFA as an alternative therapeutic approach in treating benign and malignant thyroid diseases.

## Author Contributions

All authors equally contributed to the composition of this editorial.

## Conflict of Interest

The authors declare that the research was conducted in the absence of any commercial or financial relationships that could be construed as a potential conflict of interest.

## Publisher’s Note

All claims expressed in this article are solely those of the authors and do not necessarily represent those of their affiliated organizations, or those of the publisher, the editors and the reviewers. Any product that may be evaluated in this article, or claim that may be made by its manufacturer, is not guaranteed or endorsed by the publisher.

